# Age affects the contraction-induced mitochondrial redox response in skeletal muscle

**DOI:** 10.3389/fphys.2015.00021

**Published:** 2015-02-04

**Authors:** Dennis R. Claflin, Malcolm J. Jackson, Susan V. Brooks

**Affiliations:** ^1^Department of Biomedical Engineering, University of MichiganAnn Arbor, MI, USA; ^2^Department of Surgery, Section of Plastic Surgery, University of MichiganAnn Arbor, MI, USA; ^3^Department of Musculoskeletal Biology, Institute of Ageing and Chronic Disease, University of LiverpoolLiverpool, UK; ^4^Department of Molecular and Integrative Physiology, University of MichiganAnn Arbor, MI, USA

**Keywords:** skeletal muscle, contraction, fluorescence, mitochondria, reactive oxygen species, NADH

## Abstract

Compromised mitochondrial respiratory function is associated with advancing age. Damage due to an increase in reactive oxygen species (ROS) with age is thought to contribute to the mitochondrial deficits. The coenzyme nicotinamide adenine dinucleotide in its reduced (NADH) and oxidized (NAD^+^) forms plays an essential role in the cyclic sequence of reactions that result in the regeneration of ATP by oxidative phosphorylation in mitochondria. Monitoring mitochondrial NADH/NAD^+^ redox status during recovery from an episode of high energy demand thus allows assessment of mitochondrial function. NADH fluoresces when excited with ultraviolet light in the UV-A band and NAD^+^ does not, allowing NADH/NAD^+^ to be monitored in real time using fluorescence microscopy. Our goal was to assess mitochondrial function by monitoring the NADH fluorescence response following a brief period of high energy demand in muscle from adult and old wild-type mice. This was accomplished by isolating whole lumbrical muscles from the hind paws of 7- and 28-month-old mice and making simultaneous measurements of force and NADH fluorescence responses during and after a 5 s maximum isometric contraction. All muscles exhibited fluorescence oscillations that were qualitatively similar and consisted of a brief transient increase followed by a longer transient period of reduced fluorescence and, finally, an increase that included an overshoot before recovering to resting level. Compared with the adult mice, muscles from the 28 mo mice exhibited a delayed peak during the first fluorescence transient and an attenuated recovery following the second transient. These findings indicate an impaired mitochondrial capacity to maintain NADH/NAD^+^ redox homeostasis during contractile activity in skeletal muscles of old mice.

## Introduction

Mitochondria are the main source of the adenosine triphosphate (ATP) required to support skeletal muscle contractile function. The mitochondrial processes that result in the regeneration of ATP also give rise to reactive oxygen species (ROS). If ROS levels are not sufficiently controlled by intrinsic antioxidant defense systems, oxidative damage to various cellular systems can accumulate and this process is thought to contribute to the age-related reductions in muscle mitochondrial function (Conley et al., 2000; Mansouri et al., 2006; Peterson et al., 2012).

The coenzyme nicotinamide adenine dinucleotide (NAD) in its reduced (NADH) and oxidized (NAD^+^) forms plays an essential role in the cyclic sequence of reactions that result in the regeneration of ATP by oxidative phosphorylation in mitochondria. NADH fluoresces when excited with ultraviolet (UV-A) light and NAD^+^ does not, allowing the NAD redox state to be monitored using fluorescence spectroscopy (Chance and Jobsis, 1959). Because the fluorescence captured during whole cell or tissue UV excitation is dominated by mitochondrial NADH (Mayevsky and Rogatsky, 2007), tracking the fluorescence response of skeletal muscle following a maximum tetanic contraction provides a view of mitochondrial function and mitochondrial NAD redox status during recovery from an episode of high energy demand. Moreover, since the mitochondrial NAD redox state reflects the global mitochondrial redox state (Mayevsky and Rogatsky, 2007; Zorov et al., 2014) which, in turn, is an indicator of the probability of ROS formation (Zorov et al., 2014), the fluorescence response serves as a continuous, real-time signal that predicts the development of oxidative stress.

In skeletal muscles from adult rats (Wendt and Chapman, 1976) and toads (Godfraind-De Becker, 1972), the fluorescence response following a brief isometric tetanic contraction is a large, damped oscillation that slowly returns to the pre-contraction steady-state level. Since advancing age is known to compromise mitochondrial function, we hypothesized that the mitochondrial redox response as reported by NADH fluorescence is altered in muscles from old mice following a brief period of high-intensity energy demand. Using fluorescence microscopy to monitor the redox response of isolated whole skeletal muscles from adult and old mice, we found that advanced age is associated with a change in the balance between the rates of NAD reduction and oxidation in muscle mitochondria following a brief increase in energy demand.

## Materials and methods

### Animals and operative procedure

Male wild-type mice, aged 16–17 mo (adult) or 26–33 mo (old), were obtained from the Jackson Laboratory (Bar Harbor, ME) and Dr. Holly Van Remmen at the University of Texas Health Science Center, San Antonio. Mice were deeply anesthetized with an intraperitoneal injection of tribromoethanol (Avertin, 400 mg/kg). The hind paws were removed and whole lumbrical (LMB) muscles were dissected from the medial side of digit 2 while immersed in a chilled bathing solution, composition (in mM): 137 NaCl, 11.9 NaHCO_3_, 5.0 KCl, 1.8 CaCl_2_, 0.5 MgCl_2_, 0.4 NaH_2_PO_4_. The mice were then euthanized by an overdose of the anesthetic followed by a bilateral thoracotomy. All experimental procedures were approved by the University Committee for the Use and Care of Animals at the University of Michigan and were in accordance with the Guide for Care and Use of Laboratory Animals (Public Health Service, 19965, NIH Pub. No. 85–23).

### Contractile properties

Details of the apparatus and procedures for assessing the contractile properties of isolated mouse LMB muscles have been described previously (Claflin and Brooks, 2008). Briefly, freshly dissected muscles were transferred to a chamber that was perfused at a rate of 2 exchanges/min with Tyrode solution (mM): NaCl, 121; KCl, 5.0; CaCl_2_, 1.8; MgCl_2_, 0.5; NaH_2_PO_4_, 0.4; NaHCO_3_, 24; glucose, 5.5; EDTA, 0.10. The temperature of the solution was held at 25°C and oxygenation and pH 7.3 were maintained by bubbling with a 95% O_2_, 5% CO_2_ mix. Activation was by electrical stimulation via platinum plate electrodes placed on either side of and parallel to the muscle. Muscles were mounted horizontally in the chamber with one end attached to a stationary post and the other to a force transducer (Aurora Scientific, Inc., modified Model 400A). The small size of the LMB muscle permitted visualization of sarcomere-based striations using standard brightfield microscopy. This allowed real-time monitoring of sarcomere length with a high-speed video system (model 901A, Aurora Scientific, Inc.), which was used to adjust resting sarcomere length to 2.5 μm for all experiments. The LMB muscle was then stimulated continuously for 2, 5, or 10 s at 125 pulses/s, a rate sufficient to elicit maximum isometric force. Each of the stimulus pulses was 0.2 ms in duration and exceeded the intensity required for maximum force production by approximately 25%. Force and NADH fluorescence (see below) were recorded continuously throughout the protocol.

### NADH fluorescence

The floor of the experimental chamber was made of polished quartz, which allowed unattenuated transmission of ultraviolet (UV) excitation light. The chamber was placed on the stage of an inverted microscope (Zeiss Axiovert 100). Fluorescence was elicited by epi-illumination from a 75 W xenon lamp and detected using a photomultiplier tube (Hamamatsu, model R1527 PMT). The wavelengths for excitation were centered at 361 nm (2 nm bandwidth), selected using a diffraction grating monochromator (Deltascan 4000, Photon Technology International). The emitted fluorescence passed through a 460 nm band-pass filter (50 nm bandwidth) before reaching the photomultiplier tube. Fluorescence responses were collected from a 0.23 mm by 0.92 mm rectangular area, centered on the muscle, with the long axis of the rectangle coinciding with the long axis of the muscle. Exposure of the muscle to UV light was minimized by controlling a light-blocking shutter to open only when fluorescence measurements were actively being acquired.

### Experimental procedure

**Figure 1:**
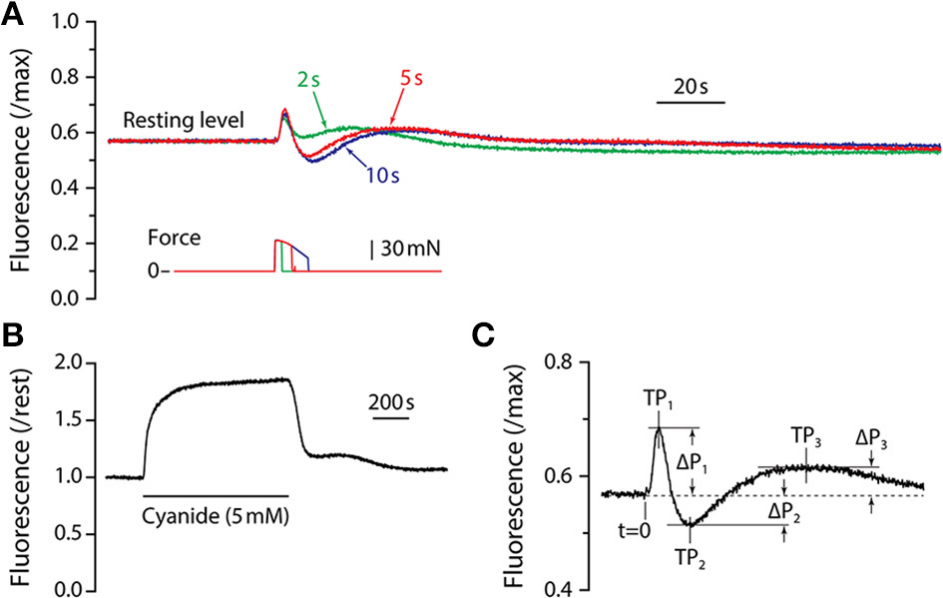
**NADH fluorescence responses**. **(A)** Fluorescence responses of adult mouse LMB muscle for tetanic contractions of 2, 5, and 10 s duration. All responses are from the same muscle and are scaled by the maximum fluorescence as determined by exposure to cyanide. Corresponding tetanic force responses are shown beneath the fluorescence traces and are plotted using the same time-base. Note that all fluorescence responses from adult muscle exhibit the same qualitative time-course consisting of a sharp, brief increase followed by an abrupt decline, then a recovery that exceeds baseline level. Fluorescence then declines to a sub-resting level before returning slowly to the pre-contraction level. **(B)** Response of LMB muscle to sodium cyanide (5 mM). The solid horizontal line beneath the fluorescence record indicates the duration of the cyanide exposure. **(C)** Parameter definitions for the first three fluorescence oscillation peaks are indicated. Peak times (TP_1_, TP_2_, TP_3_) were measured relative to *t* = 0, the time at which the contraction was initiated. Peak amplitudes (ΔP_1_, ΔP_2_, ΔP_3_) were measured relative to the pre-contraction resting level (dashed line).

After attachment to the experimental apparatus, the LMB muscle was lengthened until just taut and subjected to 3–5 single stimulus pulses of increasing amplitude, separated by 10 s. The purpose of this sequence was to determine supramaximal stimulus intensity by monitoring peak twitch forces. Sarcomere length was then set to 2.5 μm by adjusting the length of the muscle while monitoring the output of the video sarcomere length analyzer. System background fluorescence was determined by moving the microscope stage just enough to remove the muscle from the microscopic field and was subtracted from all subsequent fluorescence measurements. A minimum of 10 min was allowed to elapse between the final twitch of the sequence and any fluorescence measurements to allow NADH to return to its resting level. Simultaneous continuous recording of muscle force and fluorescence was then initiated and 3 min of baseline recording was acquired before the tetanic stimulus was applied. Following the tetanic contraction, recording continued for an additional 17 min. Sample rate was 10/s throughout. For a subset of experiments (6 of 8 adult, 4 of 10 old), the chamber perfusion was stopped and the solution was replaced with a Tyrode solution to which sodium cyanide (5 mM), an electron transport inhibitor, had been added. Introduction of the cyanide resulted in a rapid increase in fluorescence that reached a plateau level within 8–10 min (see Figure 1B). The maximum fluorescence level was interpreted as corresponding to the maximum possible ratio of NADH to total NAD (NADH + NAD^+^), or the maximally reduced mitochondrial redox state. For experiments in which the maximum fluorescence level was not determined, it was estimated using the average age-specific ratio of maximum fluorescence to resting fluorescence established in the subset of experiments in which the cyanide incubation step was carried out. Although system background fluorescence was removed from all responses (described above), no attempt was made to determine the contributions of non-NADH fluorescence through the use of uncouplers such as carbonylcyanide-p-trifluoromethoxy phenylhydrazone (FCCP) (Eng et al., 1989; Minezaki et al., 1994; Brandes and Bers, 1996; Gandra et al., 2012). Because fluorescence results are all presented after normalization by either resting or maximum fluorescence, and any non-NADH fluorescence that is present is not accounted for, non-NADH fluorescence would have the effect of reducing all reported amplitudes. The peak values, Δ-peak values, and peak-peak values presented here should therefore be considered lower-bound estimates of the true values.

### Statistics

Statistical analyses were performed using JMP software (SAS Institute, Inc.). Student's *t*-test was used to identify differences between means. Significance was set *a priori* at *p* < 0.05.

## Results

Typical fluorescence responses to maximum isometric tetanic contractions of 2, 5, and 10 s are shown in Figure 1A scaled relative to the maximum fluorescence level as determined by exposure to cyanide (Figure 1B). The earliest observed fluorescence change was a large, rapid increase that began approximately 1 s after the initiation of the contraction and reached a peak (“P_1_”) approximately 2 s later. The fluorescence intensity then reversed and fell rapidly, reaching a minimum level (“P_2_”). From the minimum, fluorescence recovered slowly to a third peak (“P_3_”), and then declined again before gradually returning to resting level. Fluorescence response amplitude (P) and time (T) parameters are defined in Figure 1C. In preliminary experiments, fluorescence responses to tetanic contraction durations of up to 20 s were recorded. Responses to all tetanus durations were qualitatively similar, exhibiting damped oscillations consisting of three distinct peaks followed by a gradual return to resting level. For contractions that were longer than 3 s, P_1_ occurred during force generation and its amplitude and timing were therefore independent of tetanus duration. The fluorescence reversal that defined P_2_ did not begin until shortly after a contraction ended, thus both its amplitude (|ΔP_2_|) and time-to-peak (T_2_) increased with contraction duration. The time required for the entire damped fluorescence oscillation to return to resting level depended on the duration of the contraction and was approximately 10 min for a 5 s tetanus and 15 min for a 10 s tetanus. Based upon these preliminary experiments, a 5 s tetanic contraction was chosen as the test condition for the remainder of the study as it resulted in a robust, characteristic fluorescence response without the large decline in force observed during longer contractions. With the exception of Figure 1A, all results reported are responses to 5 s maximum isometric tetanic contractions.

**Figure 2:**
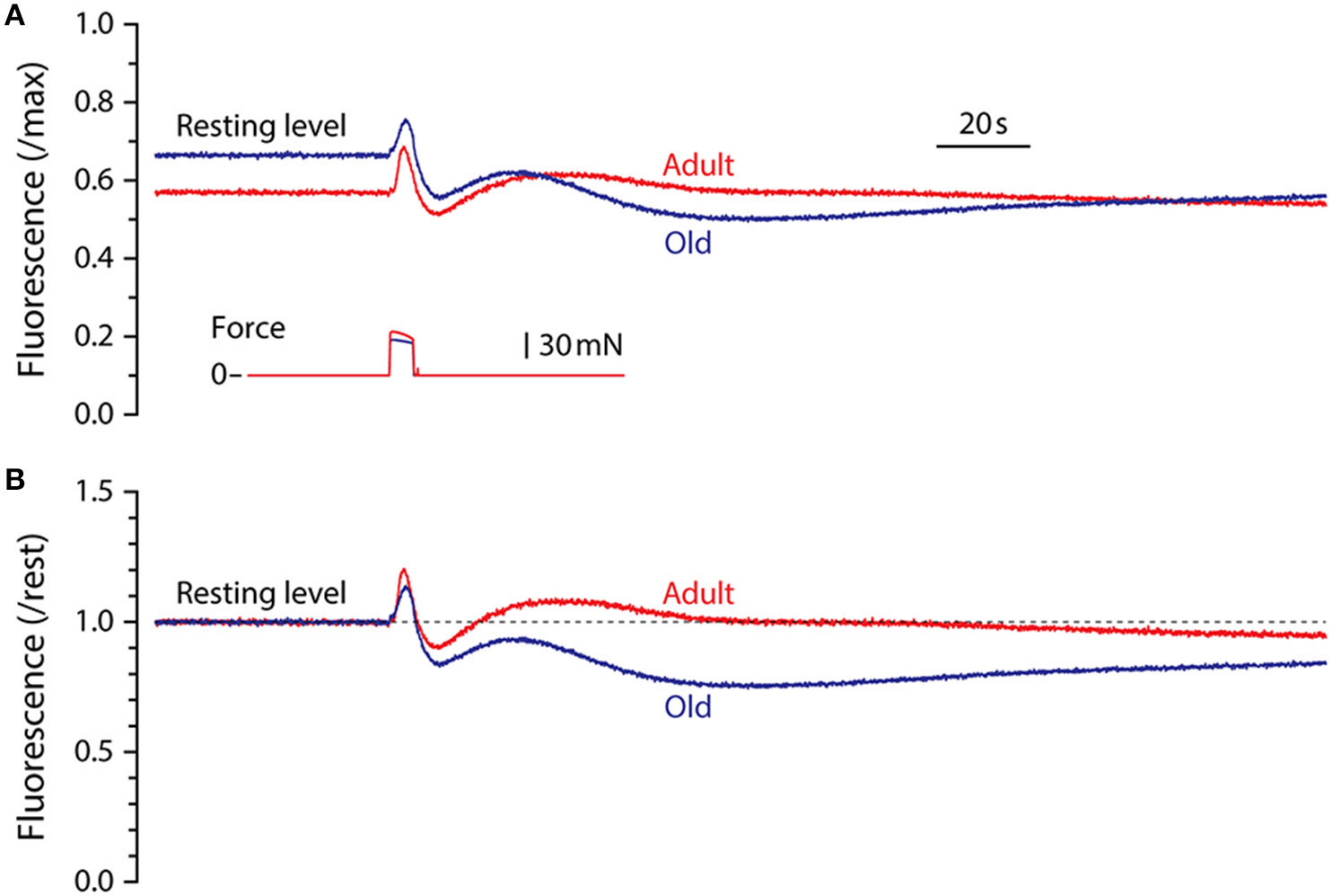
**Representative NADH fluorescence responses for LMB muscles from adult and old mice**. **(A)** Superimposed NADH fluorescence responses during 5 s maximum tetanic contractions in LMB muscles from adult and old mice. Records are scaled by the maximum fluorescence as determined by exposure to cyanide (see Figure 1B). Corresponding tetanic force responses are shown beneath the fluorescence traces. **(B)** The fluorescence responses shown in **(A)** have been re-scaled relative to pre-contraction resting levels (dashed line) to facilitate comparison of their time courses. All fluorescence and force responses shown in **(A)** and **(B)** are plotted using the same time-base.

Representative fluorescence responses to a 5 s maximum isometric tetanic contraction in LMB muscles from adult and old mice are shown in Figure 2. In Figure 2A, the fluorescence is scaled by the maximum cyanide-induced level and illustrates the finding that, relative to maximum, the resting level in muscles from old mice was higher than that in adult mice. For all experiments in which maximum fluorescence levels were determined, the resting level in muscles from adult mice, expressed as a fraction of maximum, was 0.53 ± 0.03 (mean ± SEM, *n* = 6) whereas the resting level for old mice was 0.63 ± 0.02 (*n* = 4), significantly higher (*p* = 0.026). In Figure 2B the fluorescence records shown in Figure 2A have been normalized by their respective resting levels to facilitate temporal comparisons. This representation serves to illustrate the blunted fluorescence recovery of old muscles from P_2_ to P_3_, the most striking difference observed between the responses of muscles from adult and old mice.

**Figure 3:**
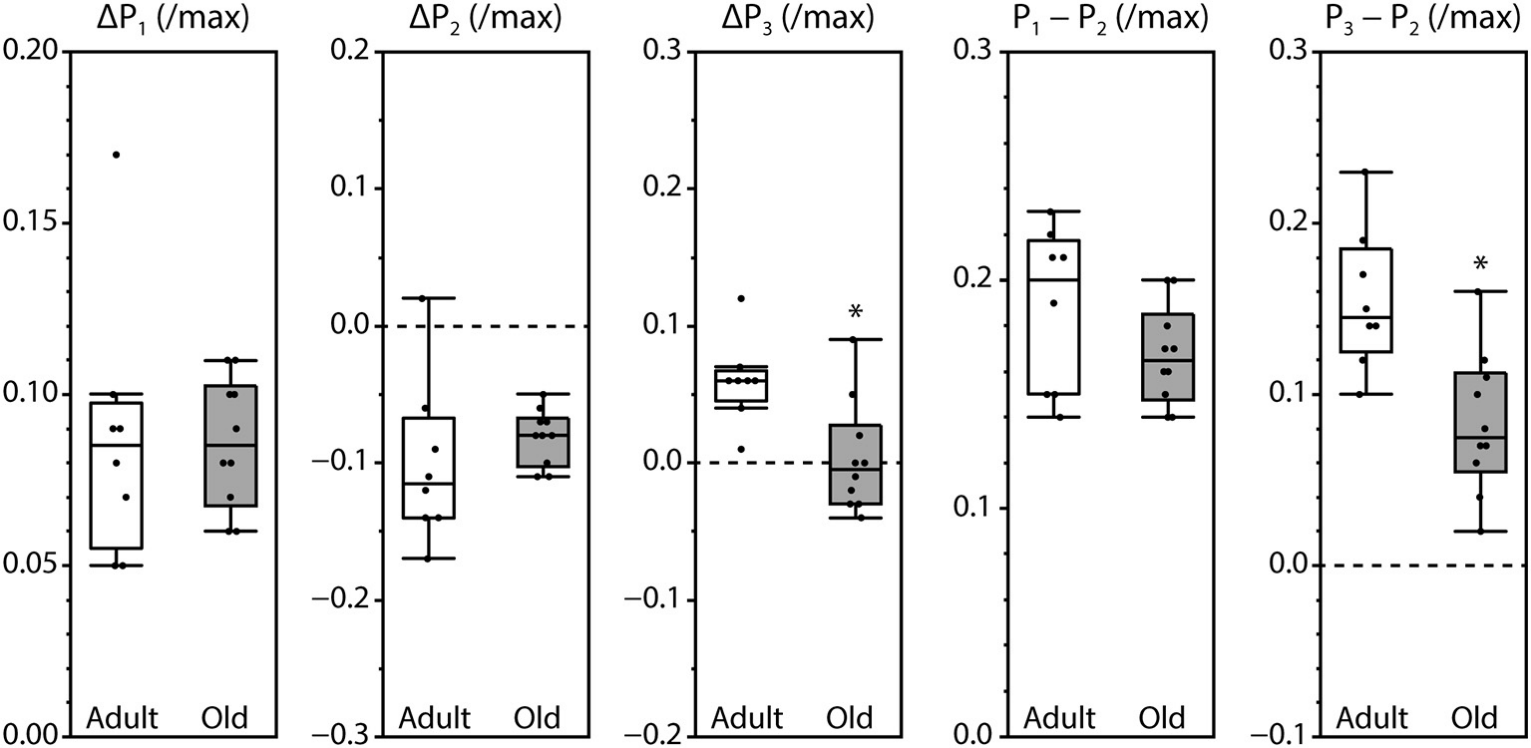
**Amplitudes of the fluorescence oscillations in LMB muscles from adult and old mice**. Definitions for the amplitude parameters are shown in Figure 1C. Box plots indicate the median and the 25th and 75th quartiles. The vertical lines that originate from the top and bottom surfaces of the boxes extend to the outermost data points that fall within 1.5× the difference between the 75th and 25th quartiles. Asterisks indicate significant differences (*p* < 0.05). Data were collected from *n* = 8 and *n* = 10 LMB muscles from adult and old mice, respectively.

**Figure 4:**
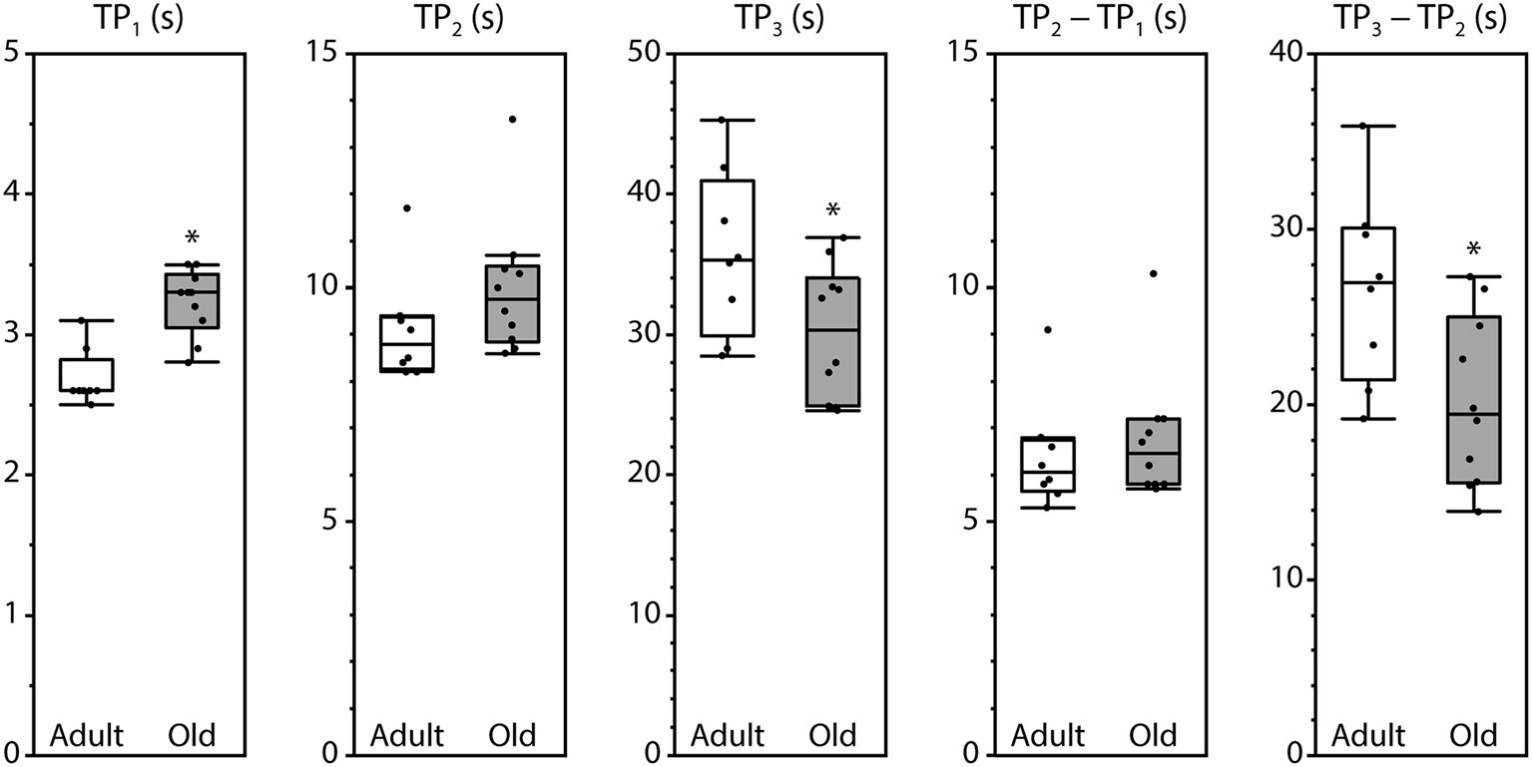
**Timing of the fluorescence oscillation peaks in LMB muscles from adult and old mice**. Definitions for the time parameters are shown in Figure 1C. Box plots indicate the median and the 25th and 75th quartiles. The vertical lines that originate from the top and bottom surfaces of the boxes extend to the outermost data points that fall within 1.5× the difference between the 75th and 25th quartiles. Asterisks indicate significant differences (*p* < 0.05). Data were collected from *n* = 8 and *n* = 10 LMB muscles from adult and old mice, respectively.

Peak fluorescence amplitudes are presented either as ΔP, the difference between resting level and peak level, or as the peak-to-peak difference between consecutive peaks, all normalized by maximum fluorescence and shown in Figure 3. Values for ΔP_1_, ΔP_2_, and P_1_–P_2_ were not affected by the age of the mouse. In contrast, ΔP_3_ and P_3_–P_2_ were significantly smaller in muscles from old mice compared with adult mice. The time intervals between the initiation of contraction and P_2_ and between P_1_ and P_2_ were not affected by age. However, the time required to reach P_1_ was increased and the time to reach P_3_ was decreased in old compared with adult, as was the time interval from P_2_ to P_3_ (Figure 4).

## Discussion

Following a brief (5 s) period of intense contractile activity by LMB muscles of mice, the NADH fluorescence response shows a rapid increase to an initial peak (P_1_), followed by a similarly rapid decline to a minimum (P_2_) that is below resting level, followed by a slower recovery to another local peak (P_3_) before gradually returning to resting level. While this response was qualitatively similar for muscles of adult and old animals, significant quantitative differences were observed. Most pronounced was the attenuation, for muscles from old mice, of the duration and magnitude of the recovery from P_2_ to P_3_. Under the conditions used in the present study, the fluorescence response is dominated by fluctuations in mitochondrial [NADH] (Mayevsky and Rogatsky, 2007). Because mitochondrial [NADH] is the net result of NAD oxidation and reduction activities, this finding indicates that, following a brief period of high energy demand, mitochondria from old mice exhibit a decreased rate of NAD^+^ reduction, an increased rate of NADH oxidation, or both. Two clear advantages of this approach support its usefulness for assessing mitochondrial function. First, the speed inherent in fluorescence measurements provides a highly resolved view of the temporal relationship between contractile activity and the dynamics of the mitochondrial response, information impossible to obtain from traditional biochemical techniques. Second, mitochondrial function is monitored within the relevant physiological environment of living contracting muscle fibers. Thus, our findings provide new insight into the effects of aging on mitochondrial function.

### Initial increase in NADH (P_1_)

The brief, early increase in mitochondrial [NADH] following tetanic contractions (P_1_) has been observed in toad skeletal muscles (Godfraind-De Becker, 1972) and in rat soleus muscles (Wendt and Chapman, 1976). Extensor digitorum longus (EDL) muscles of rats reportedly lack the initial increase in [NADH] following a tetanic contraction (Wendt and Chapman, 1976), perhaps due to differences in fiber type composition of rat soleus (predominantly type 1) and EDL (predominantly type 2) muscles (Ariano et al., 1973). The LMB muscle of the mouse hind paw contains both type 1 and type 2 fibers, although type 1 fibers occupy only ≈10% of the cross-sectional area (Smith et al., 2013). Based on this small fraction of type 1 fibers, type 2 fiber subtypes likely also contribute to the robust P_1_ response observed in the present study. P_1_ is abolished by iodoacetic acid, a specific inhibitor of glyceraldehyde-3-phosphate dehydrogenase (Godfraind-De Becker, 1972; Wendt and Chapman, 1976), indicating that the initial increase in mitochondrial [NADH] is a result of increased glycolytic activity, although the mechanism has not been established. Among the possibilities are increases in mitochondrial availability of pyruvate and/or the activity of NADH shuttle systems.

### Transitions to P_2_ and P_3_

In cardiac muscle, an abrupt increase in energy demand by myofibrillar and sarcoplasmic reticulum ATPases causes ADP levels to rise and mitochondrial [NADH] levels to fall in response to ADP-induced activation of the electron transport system (Brandes and Bers, 2002). Recovery of mitochondrial [NADH] follows, even in the presence of continued high energy demand, as the result of a delayed increase in the activity of tricarboxylic acid (TCA) cycle dehydrogenases driven by a gradually increasing mitochondrial [Ca^2+^] (Brandes and Bers, 2002). Computational models that include [ADP]-controlled modulation of NADH oxidation rates and time-delayed [Ca^2+^]-controlled modulation of NAD^+^ reduction rates by TCA cycle activity reproduce the oscillations associated with abrupt changes in energy demand in cardiac muscle (Cortassa et al., 2006). Our interpretations of the [NADH] oscillations in skeletal muscle are based, in part, upon the findings in cardiac muscle. Specifically, we attribute the rapid decline in mitochondrial [NADH] following P_1_ to ADP-induced activation of the electron transport system, resulting in an increased rate of NADH oxidation. As mitochondrial [ADP] then begins to fall due to an increase in oxidative phosphorylation, its influence on the electron transport system declines, reducing the rate of NADH oxidation by that pathway. Simultaneous with the falling NADH oxidation rate, an increase in mitochondrial [Ca^2+^] activates mitochondrial dehydrogenases, promoting an increase in the reduction of NAD^+^ to NADH by the TCA cycle. The net result of these opposing NAD oxidation and reduction activities is P_2_, where mitochondrial [NADH] reaches a nadir and begins to recover. The recovery continues to P_3_, where NADH oxidation rates again exceed NAD^+^ reduction rates and another local peak occurs.

### Response in muscles from old mice

The most striking differences between the NADH responses of muscles from adult and old mice are the declines in the duration and magnitude of the recovery from the minimum level (P_2_) to the next local maximum (P_3_). If this recovery is driven by delayed Ca^2+^-activation of TCA cycle activity as suggested by experiments on cardiac tissue (Brandes and Bers, 2002) and by computational models (Cortassa et al., 2006), then the muted response in muscles from old mice suggests diminished TCA cycle capacity or an attenuated intramitochondrial [Ca^2+^] increase in response to the preceding tetanic contraction. In support of the latter, the myoplasmic Ca^2+^ response is reduced in skeletal muscle from old humans (Delbono et al., 1995) and mice (Gonzalez et al., 2003; Umanskaya et al., 2014). Normal tetanic myoplasmic Ca^2+^ levels can be restored in old mice by overexpression of the antioxidant catalase, implicating an increase in ROS as a factor contributing to the diminished response (Umanskaya et al., 2014). Another factor that could contribute to the weak NADH recovery is increased “uncoupling” of NADH oxidation (i.e., oxidation that does not result in ATP generation), which has also been proposed to occur with aging (Conley et al., 2000; Johannsen et al., 2012). Finally, the observation that rat soleus and EDL muscles produce different NADH responses to contractions (Wendt and Chapman, 1976) raises the possibility that changes with aging in the fiber type composition contribute to the differences in NADH responses observed in the present study. We did not analyze fiber type composition of the muscles used in this study, but no changes in fiber types are observed with aging in the fore paw LMB muscle (Russell et al., manuscript under review) and minimal age-related shifts in fiber types are reported in other hind limb muscles of mice (Phillips et al., 1993; Fry et al., 2015).

A second difference between the age groups was a significant age-associated elevation in resting NADH. The observation of higher NADH for muscles of old mice is consistent with reports that the ratio of NADH to total NAD is increased in heart, lung, liver and kidney tissue from old rats (Braidy et al., 2011) and skin from older humans (Massudi et al., 2012). The elevated resting NADH indicates that the NADH-NAD^+^ pair is more reduced in old than in adult mice suggesting a more reduced mitochondrial redox environment in old mice (Mayevsky and Rogatsky, 2007; Zorov et al., 2014), although not all mitochondrial redox couples exhibit age-related changes (Dimauro et al., 2012). A more reduced mitochondrial environment in muscles of old mice increases the probability of ROS formation during cellular respiration (Zorov et al., 2014). Consistent with this possibility, mitochondria from skeletal muscles of old mice produce significantly more hydrogen peroxide (Mansouri et al., 2006), have elevated levels of regulatory proteins associated with ROS removal (Dimauro et al., 2012), and have higher antioxidant enzyme activities (Vasilaki et al., 2006) than those from adult mice. An increase in the NADH/NAD^+^ ratio is thought to be caused in part by increased activity of the NAD^+^-consuming poly (ADP-ribose) polymerase (PARP) DNA repair proteins (Braidy et al., 2011; Massudi et al., 2012), and the relative scarcity of NAD^+^ that results could have implications for other cellular enzyme systems (Sauve et al., 2006; Stein and Imai, 2012).

### Fluorescence-based NADH measurements

Monitoring mitochondrial NADH levels by measuring fluorescence offers a non-invasive means for tracking, in real-time, the mitochondrial redox state of a cell or collection of cells (Mayevsky and Rogatsky, 2007). The NADH fluorescence can thus report instantaneously the response of the mitochondrial oxidative machinery to changes in energy demand such as those that occur frequently in skeletal muscle. In addition, because the mitochondrial NADH-NAD^+^ balance is thought to reflect the global redox status of the mitochondria under study (Mayevsky and Rogatsky, 2007; Zorov et al., 2014), the fluorescence response provides valuable insights into the myriad mitochondrial functions that are affected by the redox environment and/or the availability of NADH or NAD^+^, such as calcium release by the sarcoplasmic reticulum (Zima et al., 2004), the tendency for ROS formation (Zorov et al., 2014), and the activity of sirtuin proteins (Sauve et al., 2006; Stein and Imai, 2012). Comparing fluorescence responses of skeletal muscle from adult and old mice therefore allows insights into the effects of aging on mitochondrial function and redox homeostasis and all mitochondrial processes known to be affected by redox imbalance.

### Study limitations

Recordings of mitochondrial NADH fluctuations yield the most information when the full potential of the dynamic range of the response is known. This is typically determined by applying an inhibitor of the mitochondrial electron transport chain activity such as cyanide to maximize [NADH], and then applying an “uncoupler” such as FCCP to minimize [NADH] (Eng et al., 1989; Minezaki et al., 1994; Brandes and Bers, 1996; Gandra et al., 2012). In this study maximum NADH levels were determined by application of cyanide, but no attempt was made to assess minimum levels. Thus, an increase in non-NADH fluorescence could be contributing to the increase in resting fluorescence in muscles from old mice reported. Future experiments in which NADH levels are minimized will definitively determine resting NADH fluorescence. In addition, motion can contribute undesirable artifacts during optical recordings in muscle tissue (Godfraind-De Becker, 1972; Brandes et al., 1992; Morgan et al., 1997), but only P_1_ occurs during contractile activity making TP_1_ and ΔP_1_ the only measurements potentially affected by motion. Finally, NADPH is excited and fluoresces at the same wavelengths as NADH, but the contribution of NADPH fluorescence to total tissue fluorescence is reported to be small (Mayevsky and Rogatsky, 2007).

## Summary/conclusions

We have shown that, compared with adult mice, the resting fluorescence level and dynamic fluorescence response to an energetic challenge differ substantially in skeletal muscles of old mice. The differences suggest an aging-associated hyper-reduced cellular environment and blunted mitochondrial NADH recovery.

### Conflict of interest statement

The authors declare that the research was conducted in the absence of any commercial or financial relationships that could be construed as a potential conflict of interest.

## References

[B1] Ariano M. A. Armstrong R. B. Edgerton V. R. (1973). Hindlimb muscle fiber populations of five mammals. J. Histochem. Cytochem. 21, 51–55. 4348494 10.1177/21.1.51

[B2] Braidy N. Guillemin G. J. Mansour H. Chan-Ling T. Poljak A. Grant R. (2011). Age related changes in NAD^+^ metabolism oxidative stress and Sirt1 activity in wistar rats. PLoS ONE 6:e19194. 10.1371/journal.pone.0019194 21541336 PMC3082551

[B3] Brandes R. Bers D. M. (1996). Increased work in cardiac trabeculae causes decreased mitochondrial NADH fluorescence followed by slow recovery. Biophys. J. 71, 1024–1035. 10.1016/S0006-3495(96)79303-7 8842239 PMC1233557

[B4] Brandes R. Bers D. M. (2002). Simultaneous measurements of mitochondrial NADH and Ca^2+^ during increased work in intact rat heart trabeculae. Biophys. J. 83, 587–604. 10.1016/S0006-3495(02)75194-1 12124250 PMC1302172

[B5] Brandes R. Figueredo V. M. Camacho S. A. Massie B. M. Weiner M. W. (1992). Suppression of motion artifacts in fluorescence spectroscopy of perfused hearts. Am. J. Physiol. 263, H972–H980. 1415626 10.1152/ajpheart.1992.263.3.H972

[B6] Chance B. Jobsis F. F. (1959). Changes in fluorescence in a frog sartorius muscle following a twitch. Nature 184, 195–196 10.1038/184195a0

[B7] Claflin D. R. Brooks S. V. (2008). Direct observation of failing fibers in muscles of dystrophic mice provides mechanistic insight into muscular dystrophy. Am. J. Physiol. Cell Physiol. 294, C651–C658. 10.1152/ajpcell.00244.2007 18171725

[B8] Conley K. E. Jubrias S. A. Esselman P. C. (2000). Oxidative capacity and ageing in human muscle. J. Physiol. 526(Pt 1), 203–210. 10.1111/j.1469-7793.2000.t01-1-00203.x 10878112 PMC2269983

[B9] Cortassa S. Aon M. A. O'rourke B. Jacques R. Tseng H. J. Marban E.. (2006). A computational model integrating electrophysiology, contraction, and mitochondrial bioenergetics in the ventricular myocyte. Biophys. J. 91, 1564–1589. 10.1529/biophysj.105.076174 16679365 PMC1518641

[B10] Delbono O. O'rourke K. S. Ettinger W. H. (1995). Excitation-calcium release uncoupling in aged single human skeletal muscle fibers. J. Membr. Biol. 148, 211–222. 8747553 10.1007/BF00235039

[B11] Dimauro I. Pearson T. Caporossi D. Jackson M. J. (2012). *In vitro* susceptibility of thioredoxins and glutathione to redox modification and aging-related changes in skeletal muscle. Free Radic. Biol. Med. 53, 2017–2027. 10.1016/j.freeradbiomed.2012.09.031 23022873 PMC3657158

[B12] Eng J. Lynch R. M. Balaban R. S. (1989). Nicotinamide adenine dinucleotide fluorescence spectroscopy and imaging of isolated cardiac myocytes. Biophys. J. 55, 621–630. 10.1016/S0006-3495(89)82859-0 2720061 PMC1330544

[B13] Fry C. S. Lee J. D. Mula J. Kirby T. J. Jackson J. R. Liu F.. (2015). Inducible depletion of satellite cells in adult, sedentary mice impairs muscle regenerative capacity without affecting sarcopenia. Nat. Med. 21, 76–80. 10.1038/nm.3710 25501907 PMC4289085

[B14] Gandra P. G. Nogueira L. Hogan M. C. (2012). Mitochondrial activation at the onset of contractions in isolated myofibres during successive contractile periods. J. Physiol. 590, 3597–3609. 10.1113/jphysiol.2012.232405 22711953 PMC3547273

[B15] Godfraind-De Becker A. (1972). Heat production and fluorescence changes of toad sartorius muscle during aerobic recovery after a short tetanus. J. Physiol. 223, 719–734. 4339903 10.1113/jphysiol.1972.sp009871 PMC1331478

[B16] Gonzalez E. Messi M. L. Zheng Z. Delbono O. (2003). Insulin-like growth factor-1 prevents age-related decrease in specific force and intracellular Ca^2+^ in single intact muscle fibres from transgenic mice. J. Physiol. 552, 833–844. 10.1113/jphysiol.2003.048165 12937290 PMC2343464

[B17] Johannsen D. L. Conley K. E. Bajpeyi S. Punyanitya M. Gallagher D. Zhang Z.. (2012). Ectopic lipid accumulation and reduced glucose tolerance in elderly adults are accompanied by altered skeletal muscle mitochondrial activity. J. Clin. Endocrinol. Metab. 97, 242–250. 10.1210/jc.2011-1798 22049170 PMC3251940

[B18] Mansouri A. Muller F. L. Liu Y. Ng R. Faulkner J. Hamilton M.. (2006). Alterations in mitochondrial function, hydrogen peroxide release and oxidative damage in mouse hind-limb skeletal muscle during aging. Mech. Ageing Dev. 127, 298–306. 10.1016/j.mad.2005.11.004 16405961

[B19] Massudi H. Grant R. Braidy N. Guest J. Farnsworth B. Guillemin G. J. (2012). Age-associated changes in oxidative stress and NAD^+^ metabolism in human tissue. PLoS ONE 7:e42357. 10.1371/journal.pone.0042357 22848760 PMC3407129

[B20] Mayevsky A. Rogatsky G. G. (2007). Mitochondrial function *in vivo* evaluated by NADH fluorescence: from animal models to human studies. Am. J. Physiol. Cell Physiol. 292, C615–C640. 10.1152/ajpcell.00249.2006 16943239

[B21] Minezaki K. K. Suleiman M. S. Chapman R. A. (1994). Changes in mitochondrial function induced in isolated guinea-pig ventricular myocytes by calcium overload. J. Physiol. 476, 459–471. 8057254 10.1113/jphysiol.1994.sp020147 PMC1160460

[B22] Morgan D. L. Claflin D. R. Julian F. J. (1997). The relationship between tension and slowly varying intracellular calcium concentration in intact frog skeletal muscle. J. Physiol. 500, 177–192. 9097942 10.1113/jphysiol.1997.sp022008 PMC1159368

[B23] Peterson C. M. Johannsen D. L. Ravussin E. (2012). Skeletal muscle mitochondria and aging: a review. J. Aging Res. 2012, 194821. 10.1155/2012/194821 22888430 PMC3408651

[B24] Phillips S. K. Wiseman R. W. Woledge R. C. Kushmerick M. J. (1993). Neither changes in phosphorus metabolite levels nor myosin isoforms can explain the weakness in aged mouse muscle. J. Physiol. 463, 157–167 8246180 10.1113/jphysiol.1993.sp019589 PMC1175338

[B25] Sauve A. A. Wolberger C. Schramm V. L. Boeke J. D. (2006). The biochemistry of sirtuins. Annu. Rev. Biochem. 75, 435–465. 10.1146/annurev.biochem.74.082803.133500 16756498

[B26] Smith I. C. Gittings W. Huang J. McMillan E. M. Quadrilatero J. Tupling A. R.. (2013). Potentiation in mouse lumbrical muscle without myosin light chain phosphorylation: is resting calcium responsible? J. Gen. Physiol. 141, 297–308. 10.1085/jgp.201210918 23401574 PMC3581688

[B27] Stein L. R. Imai S. (2012). The dynamic regulation of NAD metabolism in mitochondria. Trends Endocrinol. Metab. 23, 420–428. 10.1016/j.tem.2012.06.005 22819213 PMC3683958

[B28] Umanskaya A. Santulli G. Xie W. Andersson D. C. Reiken S. R. Marks A. R. (2014). Genetically enhancing mitochondrial antioxidant activity improves muscle function in aging. Proc. Natl. Acad. Sci. U.S.A. 111, 15250–15255. 10.1073/pnas.1412754111 25288763 PMC4210348

[B29] Vasilaki A. McArdle F. Iwanejko L. M. McArdle A. (2006). Adaptive responses of mouse skeletal muscle to contractile activity: the effect of age. Mech. Ageing Dev. 127, 830–839. 10.1016/j.mad.2006.08.004 16996110

[B30] Wendt I. R. Chapman J. B. (1976). Fluorometric studies of recovery metabolism of rat fast- and slow-twitch muscles. Am. J. Physiol. 230, 1644–1649 937554 10.1152/ajplegacy.1976.230.6.1644

[B31] Zima A. V. Copello J. A. Blatter L. A. (2004). Effects of cytosolic NADH/NAD^+^ levels on sarcoplasmic reticulum Ca^2+^ release in permeabilized rat ventricular myocytes. J. Physiol. 555, 727–741. 10.1113/jphysiol.2003.055848 14724208 PMC1664876

[B32] Zorov D. B. Juhaszova M. Sollott S. J. (2014). Mitochondrial reactive oxygen species (ROS) and ROS-induced ROS release. Physiol. Rev. 94, 909–950. 10.1152/physrev.00026.2013 24987008 PMC4101632

